# Supplementary material for: Testing the skill-based approach: Consolidation strategy impacts attentional blink performance
Source: PLoS One. 2022 Jan 21;17(1):e0262350. doi: 10.1371/journal.pone.0262350 (PMC8782399; doi:10.1371/journal.pone.0262350)
Supplement: Attachment — Submitted filename: Response to reviewers.docx [file pone.0262350.s001.docx]

Dear Dr. Livesey,

Thank you for considering our manuscript for publication in PLOS ONE. We appreciate the constructive comments provided by you and the reviewers and they have led to an improved manuscript. In our revision we have focused on modifying the conclusions we draw to reflect more uncertainty and include other potential explanations. Additionally, we have clarified certain ambiguities and added several important details that were missing in the original manuscript. We hope that these modifications will be sufficient to address the concerns raised by you and the reviewers. Below you can find a more detailed response to the points raised in the review. All page and line numbers refer to the revised manuscript with tracked changes.

**Editor remarks:**

1. You and the reviewers note that the model predictions were only linked to the data *post hoc* and that there is no clear explanation for Cluster 3. We agree with these concerns and we have added several paragraphs to the manuscript to clarify and reflect these limitations. Regarding the *post hoc* nature of the model fit we have added lines 908-922 acknowledging the post hoc nature and suggest an experiment which might test the hypotheses generated by the cluster analysis. Additionally, we have added lines 898-907 elaborating on Cluster 3 and present a possible explanation for the performance pattern in this cluster. Furthermore, lines 799-803 also touch on the possibility that participants might spontaneously adopt other cognitive strategies than we and the model assumed.
2. The second point concerns the high T2 accuracy in Cluster 1 and whether this can be taken as evidence for our model. We second that the high performance by such a large group of participants is very surprising and contrasts with the common conception of the AB as a fundamental limit of cognition. In the paragraph on lines 877-881 we present the argument for the case that the different clusters arise from employing different strategies (in essence, Cluster 1 and 2 show qualitatively different performance patterns doing the same task with the same stimuli. Which we think is unlikely to reflect fundamental different levels of individual ability, especially because many previous AB tasks have shown that people are very limited in their ability to identify two rapidly present successive targets). The final two paragraphs of the discussion of Experiment 2 (p. 37-38) are rewritten and reflect the uncertainty surrounding any hard conclusions about the mechanisms responsible for our results. The first paragraph (lines 908-922) discusses the uncertainty surrounding what these strategies exactly entail and that our data cannot unquestionably show that they are the two strategies we predicted. Furthermore, in the final paragraph (line 923-942) the final conclusion has been modified to reflect the uncertainty in crediting any explicit mechanism for the reduced AB. Furthermore, in this conclusion we repeat the two factors underlying our decision to perform a cluster analysis and the post hoc nature of this analysis.
3. Finally, we have added the missing details you mentioned. The font is mentioned on lines 324-325 and 461-462. The single target trials are analyzed on lines 694-697. The proportion of omitted trials are reported on line 535. Furthermore, while analyzing the proportion of omitted trials, we noticed that the number of excluded trials was very high for two participants. These participants have now been removed completely and the outcomes of the statistics we ran and the figures have been adjusted throughout the manuscript. This did not significantly change the results; the main change was that Cluster 3 included two participants less, as both were previously assigned to that cluster.

**Reviewer 1**

1. The first point raised by the reviewer states that our replication also might have failed because the results are not replicable at all. We agree with this point and have added this possibility to the discussion of Exp. 1 (p. 17).
2. The second point has been largely discussed in point 2 of the Editor remarks. It relates to the fact that the high performance in Cluster 1 could also have been caused by something else than the ‘consolidate-chunked’ strategy. We agree with this point and we realize that we were too strong in our conclusions in the original manuscript. We have moderated the conclusions in several parts of the revised manuscript to better reflect the uncertainty (e.g., on lines 887-890 and p. 40).

**Reviewer 2**

1. The first point raised by reviewer 2 concerns the post hoc nature of the model fit. We have discussed this concern in the first point of the Editor remarks and, in addition to the two previously mentioned paragraphs we have slightly changed the phrasing on line 797 as suggested by the reviewer.
2. The second point concerned a request to change Fig. 8 for it to be easier to read by thickening the lines representing the condition averages, which has been done accordingly.

In closing, we would like to thank you for your attention to our manuscript, and we hope that it now meets the publication criteria of PLOS ONE. We look forward to your response.

With kind regards,

Corné Hoekstra

Sander Martens

Niels Taatgen
